# Sjögren’s patient subgroups identified through whole genome DNA methylation profiling

**DOI:** 10.1186/s13075-026-03744-7

**Published:** 2026-02-04

**Authors:** Olivia Solomon, Caroline Shiboski, Kimberly E. Taylor, Hong Quach, Diana Quach, Lisa F. Barcellos, Lindsey A. Criswell

**Affiliations:** 1 Genetic Epidemiology and Genomics Laboratory, School of Public Health, University of California, 324 Stanley Hall, MC#3220, Berkeley, CA 94720 USA; 2 Center for Computational Biology, Division of Computing, Data Science and Society, University of California, Berkeley, California USA; 3 Department of Orofacial Sciences, School of Dentistry, University of California, San Francisco, California USA; 4 Department of Medicine, Russell/Engleman Rheumatology Research Center, University of California, San Francisco, California USA; 5 Genomics of Autoimmune Rheumatic Disease Section, National Human Genome Research Institute, National Institute of Health, Bethesda, Maryland USA

## Abstract

**Objective:**

Sjögren’s disease (SjD) is a heterogeneous autoimmune disorder characterized by lymphocytic infiltration of exocrine glands resulting in severe oral and ocular dryness. Previous published work showed DNA methylation (DNAm) can distinguish SjD case subgroups based on clinical features; however, studies used small samples and did not adjust for cellular heterogeneity in labial salivary glands (LSGs). Our objectives were to: (1) identify DNAm clusters from LSGs; (2) investigate cluster clinical characteristics; and (3) identify differential methylation between SjD case subgroups to further understand biological pathways.

**Methods:**

We identified clinically meaningful subgroups of SjD through hierarchical clustering of DNAm embeddings from a variational autoencoder (VAE) of LSGs, which allows for a low dimensional representation of the high dimensional methylation data. LSGs from 1,059 individuals, SjD cases (*n* = 592) and symptomatic non-cases (*n* = 467) were profiled using the Illumina HumanMethylationEPIC BeadChip, and cell-type proportions were estimated using a solid-tissue reference.

**Results:**

Participants clustered into subgroups with differential SjD features and proportions of predicted cell-types. Comparison of SjD cases within distinct clusters showed evidence for differential methylation in each cell-type. The largest number of differences between subgroups occurred in epithelial and B-cells and were in genes and pathways with relevance to disease pathogenesis. In B-cells, methylation within *NR2F2*, previously reported to be differentially expressed in lacrimal glands of SjD mouse models, and *NDRG2*, which increases saliva production in estrogen deficient rats, distinguished subgroups with different clinical manifestations. Additional candidates of interest identified in epithelial and B-cells from SjD cases include genes previously implicated in systemic lupus erythematosus.

**Conclusion:**

These findings provide insight into the powerful link between epigenetics and clinical heterogeneity in SjD and contribute to classification of important patient subgroups.

**Supplementary Information:**

The online version contains supplementary material available at 10.1186/s13075-026-03744-7.

## Introduction

Sjögren’s disease (SjD) is a multisystem autoimmune disorder characterized by lymphocytic infiltration of exocrine glands resulting in severe oral and ocular dryness and frequent complaints of fatigue and arthralgia [1]. It is the second most common systemic autoimmune disorder in the United States after rheumatoid arthritis, with a female-to-male ratio of 14:1 [2–5]. SjD has a heterogeneous clinical presentation; however, while definitive classification criteria for SjD have been endorsed by the American College of Rheumatology (ACR) and the European League Against Rheumatism (EULAR), formal disease subgroup-specific classification criteria have not been developed. The current 2016 ACR-EULAR classification criteria are used to classify cases based on the sum of weighted scores from five criteria items that include anti-SSA(Ro) antibody serology, labial salivary gland (LSG) histology, and objective tests of ocular and oral dryness [6]. 

Previous studies have used multiple data sources in attempt to further classify SjD patient subgroups. Autoantibody status has also been associated with clinical measures; however, the utility to predict patient subgroups is limited [7]. Using clustering, the United Kingdom Primary Sjögren’s Syndrome Registry stratified SjD cases according to self-reported symptoms of depression, anxiety, pain, fatigue, and dryness and found differential treatment effects [8]. Another study combined patient-reported symptoms with autoantibody and additional biological data for unsupervised clustering and found that individuals clustered into three groups: B-cell activated disease with low symptom burden; high systemic disease activity; and low systemic disease activity with high symptom burden [9]. The clustering classification based on symptoms and clinical and biological manifestations did not correlate with the previous symptom only classification. However, results suggest that the use of biological, clinical, and patient reported data has high utility for identifying SjD subgroups [9]. Further work is needed to characterize the underlying biological mechanisms responsible for these differences.

Bulk tissue such as LSGs consists of different cell-types which have individual epigenetic profiles. Current methods for reference-based cell-type estimation using methylation data allow for estimation of nine cell-types in solid tissue from three categories: epithelial cells, fibroblasts, and seven immune cell subtypes. Importantly, different cell-types also play individual roles in disease pathogenesis. In particular, dendritic, monocyte, epithelial, CD4 + T, CD8 + T, and B-cells have defined roles in SjD pathogenesis [10]. 

DNA methylation (DNAm) has been studied in association with SjD to better understand disease pathogenesis. Studies have reported differential methylation in SjD cases compared to healthy controls in whole blood, immune cells, and in LSG biopsies, providing strong evidence for a role of epigenetics in disease pathogenesis [11–21]. To determine if DNAm profiling could also differentiate patient subgroups, a recent study in a subset of the Sjögren’s International Collaborative Clinical Alliance (SICCA) registry used genome-wide DNAm data to cluster participants [22]. The study revealed more clinically severe and mild subgroups of SjD with a pattern of *hypomethylation* in the major histocompatibility complex (MHC) and *hypermethylation* in other areas of the genome distinguishing the subgroups [21]. However, this study was small (*n* = 131) and did not account for cellular heterogeneity among participants.

In the current study, among a large subset of 1,059 SICCA registry participants including both cases and non-cases of SjD, our objectives were to: (1) identify DNAm clusters from LSGs; (2) investigate cluster clinical characteristics; and (3) identify differential methylation between SjD case subgroups, to further understand biological pathways. Additionally, we aimed to estimate and adjust for cell-type heterogeneity, and performed cell-specific analyses to determine whether observed differences were due to cell proportion differences, and in which cell-types differential methylation was associated with clinical differences.

## Materials and methods

### Study population and clinical evaluation

Study participants included 592 SjD cases and 467 non-cases from the SICCA registry. While SICCA participants were recruited from 9 international sites in 7 countries including the U.S., and included both women and men, we restricted our study population to women of either non-Hispanic White or East Asian ancestry, who represented the largest subgroups in the registry, and without other autoimmune, systemic, or connective tissue diseases. Non-cases were deemed “symptomatic” because eligibility criteria for the SICCA registry required participants, in addition to being 21 years or older, to exhibit *at least one* of the following: symptoms of dry eyes or dry mouth, prior suspicion/diagnosis of SjD, positive serum anti-Ro/SSA, anti-La/SSB, positive rheumatoid factor or an elevated antinuclear antibody titer, sudden increase in dental caries, and bilateral parotid gland enlargement [23]. 

SjD case status was determined according to the 2016 ACR/EULAR classification criteria that are based on the weighted sum of five criteria items: anti-SSA(Ro) antibody positivity and focal lymphocytic sialadenitis (FLS) with focus score (FS) ≥ 1 foci/mm^2^ in LSG biopsies, each scoring 3; ocular staining score (OSS) ≥ 5, Schirmer test ≤ 5 mm/5 min, and unstimulated whole saliva (UWS) flow rate ≤ 0.1 mL/min, each scoring 1. The maximum score is nine; individuals with scores greater than or equal to four are classified as having SjD [6]. In addition to salivary, oral, ocular, and serological test assessments that were used to classify participants as cases and non-cases, we also obtained detailed demographic and phenotypic data on all participants from the SICCA registry. Data collection was standardized through training and calibration protocols involving all SICCA sites.

The SICCA study was approved by the Institutional Review Board of the Human Research Protection Program at the University of California, San Francisco, and from each of the other international and domestic research sites. Written informed consent was obtained from each participant.

### Methylation and preprocessing

LSGs were flash-frozen and stored in liquid nitrogen following standardized procedures at time of enrollment. LSGs of similar sizes were processed for DNA extraction using a standardized protocol. DNA methylation was measured for each LSG using the Infinium MethylationEPIC V1 (EPIC) platform.

Methylation data were processed using the *Minfi* Bioconductor package [24]. Starting with 865,859 CpG sites, background subtraction with dye-bias correction was performed using *noob* normalization and data were quantile normalized using *preprocessQuantile* [25]. Probes where more than 5% of samples had a detection *p*-value > 0.01, probes near SNPs in European and East Asian populations, and cross-reactive and polymorphic probes identified by McCartney et al. were filtered [26]. Sample filtering included removing samples with more than 5% of probes with a detection *p*-value > 0.01. The final dataset consisted of 739,659 CpG sites and 1,059 participants.

Methylation measures of β-values and M-values were used for analysis. A β-value ranges from 0 to 1, is defined as the ratio of the methylated probe intensity to the sum of methylated and unmethylated probe intensities and is interpreted as the proportion of methylation at a CpG site. The M-value is unbounded and is defined as a logit transformation of the β-value as $\:{\mathrm{log}}_{2}\frac{\beta}{1-\beta}$. The M-value is less heteroscedastic and more closely meets normality assumptions for regression analysis [27]. 

We used *HEpiDISH* to estimate cell-type proportions for each LSG sample [28]. This hierarchical method first uses a solid tissue reference to estimate total proportions of epithelial cells, fibroblasts, and immune cells. It is then reapplied to the immune cell portion to estimate the fractions of seven immune cell-types including B-cells, monocytes, CD8 + T cells, CD4 + T cells, NK cells, neutrophils, and eosinophils. The resulting cell-type proportions which can be included in multivariable regression models.

### Variational autoencoder summary

We used a variational autoencoder (VAE) to learn a compressed, lower-dimensional representation of the high-dimensional methylation data [29]. VAEs were developed in 2013 and have been widely applied in both computer science and starting in 2018, have been used in analysis of epigenomic data including DNA methylation in SjD and gene expression data in studies of cancer and lung cancer [21, 30, 31]. A VAE consists of an encoding stage and a decoding stage. The encoder part of the VAE maps the input methylation data to a lower-dimensional latent space, which serves as a compressed representation of the original data; the decoder part of the VAE maps points from the latent space back to the data space, generating reconstructions of the input methylation data.

VAEs have several desirable properties. First, the encoder and decoder can be parameterized by neural networks, which can cope with the high-dimensional methylation data. Second, the compressed latent state learned by the VAE is typically smooth—differences in the latent space reflect similar distances in DNA methylation. This smoothness combined with the low dimensionality of the latent space enables effective clustering of the compressed methylation data. We used the VAE implementation Tybalt, with hyperparameters as previously described [21, 30]. Briefly, the most relevant implementation details include: a 90% split of the data for training and 10% for validation; the VAE was applied to the top 100,000 most variable CpG sites by median absolute deviation; and the VAE neural network was trained with batch size 16 for 50 epochs. CpG sites were adjusted by regressing out sample plate, age, smoking status, and genetic ancestry (determined by cluster membership in PCA of genetic data) prior to input to the VAE.

### Hierarchical clustering

Clustering was performed using the R package *ConsensusClusterPlus*, which uses repeated sampling (*n* = 1,000) to provide clusters with more stable memberships [32]. This approach ensures the observed clusters are stable and reproducible across subsets of the data and is a robust clustering method. Euclidian distance between latent features was clustered with hierarchical clustering using the Ward’s minimum variance method as the link function [33]. Ward’s method merges the pair of clusters that results in the minimum increase in within-cluster variance at each iteration.

### Identification of differentially methylated regions: LSGs

*Bumphunter* was used to identify differentially methylated regions (DMRs) between SjD case subgroups defined by initial clustering as further described in the Results [34]. A DMR was required to have at least two CpG sites and have an effect size of greater than or equal to one, and a family-wise error rate (*fwerArea*) less than or equal to 0.05. DMRs were identified using models adjusted and unadjusted for cell-type proportions—all models adjusted for age, race, ever/never smoking status, and sample plate. Parameters for *bumphunter* included *B* = 1,000 permutations and *nullMethod* = “bootstrap”. We used *minfi* to annotate each significant DMR to its nearest gene and location relative to the nearest CpG island which contains a high concentration of CpG sites and is often found in gene promoters.

### Identification of differentially methylated regions: cell-specific

We used *CellDMC* to perform differential methylation analysis within predicted cell-types from *HEpiDISH* [35]. *CellDMC* can determine specific cell-type(s) driving differential methylation at a site. As this tool is only able to generate statistics for single CpG sites, we then used Comb-p, an alternative DMR discovery tool which requires only *p*-values from a single site analysis, to perform a regional analysis using spatially correlated p-values [36]. Parameters for comb-p included *dist.cutoff =* 1,000, *bin.size =* 310, and *seed = 0.001*. We required a DMR to have at least two CpG sites and a Dunn-Sidak *p*-value less than or equal to 0.05.

### Gene set enrichment analysis

We selected genes with significantly associated DMPs and DMRs in the promoter or gene body, where differential methylation may be more likely to contribute to changes in gene expression, for gene set enrichment analysis (GSEA) [37]. To provide a qualitative picture of the biological processes impacted by differential methylation, DMP and DMR genes were tested for enrichment of gene ontology (GO) gene sets from the Molecular Signatures Database combined with SjD-related gene sets from past studies using the hypergeometric test [13, 38, 39]. GSEA was conducted using *missMethyl* adjusting for bias due to number of probes included per gene on the EPIC array [40]. GSEA was performed separately for *hypermethylated* and *hypomethylated* genes.

The dataset supporting the conclusions of this article is available through GEO, [Accession number TBD].

## Results

### Identification of SICCA participant subgroups

**Fig. 1:**
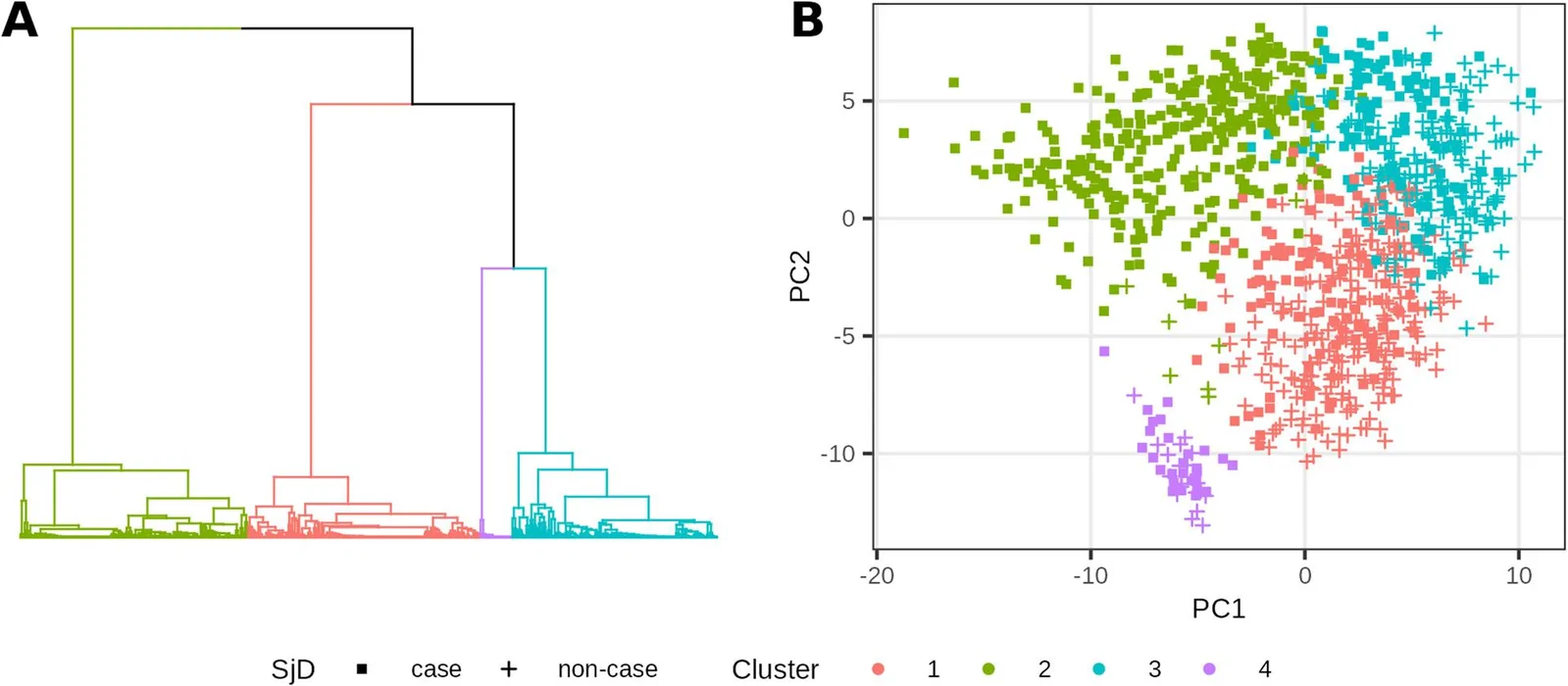
**A** Dendrogram of hierarchical clustering of VAE embeddings from all participants (**B**) PCA plot of VAE embeddings for all participants, with cluster numbering denoted by color and SjD status denoted by shape. Under repeated subsampling mean within-cluster consensus values ranged from 0.78 to 0.97 across clusters and between-cluster consensus values ranged between 0.008–0.13

We initially identified *four* distinct clusters of participants (total *N* = 1,059) through hierarchical clustering of the VAE embeddings of the most variable 100,000 CpG sites. We used SjD cases and symptomatic non-cases to identify clusters based on clinical manifestations. VAE training can be visualized in Supplementary Fig. 1. Mean within-cluster consensus values ranged from 0.78 to 0.97 across clusters, indicating high reproducibility of sample co-assignment under repeated subsampling. Between-cluster consensus values were uniformly low (0.008–0.13), indicating minimal ambiguity between clusters. The cluster dendrogram and PCA visualization of these clusters can be found in Fig. 1. Notably, clusters 2 and 4 were comprised of a *majority of SjD cases* (92% cases). Clusters 1 and 3 were comprised of a *majority of non-cases* (65% non-cases); however, some SjD cases were assigned to the clusters comprised primarily of non-cases. Further assessment of the characteristics of the participants in each of these four clusters showed that the SjD cases, specifically, in clusters 2 and 4 had more phenotypic features that reflected higher levels of autoimmunity and higher frequency of SjD-related clinical manifestations compared to SjD cases assigned to clusters 1 and 3 (Table 1). Overall, each of the five individual criteria items comprising the ACR/EULAR score and some other phenotypic characteristics were observed more frequently in the cases who were assigned to clusters 2 and 4. The proportions of high focus scores (mean of 1.3 vs. 3.7, *p* < 0.01), ANA autoantibody positivity (at a concentration of 1:320), lower levels of complement components 3 and 4, anti-La/SSB positivity, rheumatoid factor positivity, ocular SICCA score, right and left parotid gland enlargement, and subjects with germinal center formation detected on LSG biopsies by H&E were all significantly higher in the SjD cases present in clusters 2 and 4. Although self-report dry mouth and dry eye symptoms were also reported more frequently for cases in clusters 2 and 4, these differences did not reach statistical significance. Because the majority of SjD cases were observed in clusters 2 and 4, and the majority of non-cases were observed in clusters 1 and 3, our analyses then focused on comparisons between two subgroups of SjD cases; those cases *only* in clusters 2 and 4 (herein subgroup 2–4; *N* = 358) and those cases *only* in clusters 1 and 3 (herein subgroup 1–3; *N* = 234). Symptomatic non-cases were excluded from these analyses. Additionally, age, self-reported race/ethnicity, and smoking history were significantly different between subgroup 2–4 and subgroup 1–3 and were therefore adjusted for in all downstream analyses. Results of analyses stratified by self-reported race/ethnicity are reported in Supplementary Tables 13–20. The proportion of patients in SjD case subgroups who reported taking immunomodulating drugs did not differ. This includes corticosteroids (*p* = 0.5), alkylating agents (*p* = 0.8), antimetabolites (*p* = 1.0), TNF-alpha inhibitors (*p* = 0.7), other disease-modifying antirheumatic drugs (*p* = 1.0), antimalarials (*p* = 0.1), anti-CD-20 (*p* = 0.9), and other immune modifying drugs (*p* = 1.0). When excluding non-cases from clustering subgroup membership 95.4% of cases remained in the same subgroup. Results are summarized in Table 1 separated by SjD case/non-case, individual clusters, and subgroup 2–4 and subgroup 1–3. Correlations among clinical features and cell-type proportions were examined and results are shown in Supplementary Fig. 3. As expected, modest correlations (> 0.45) were observed for some clinical variables including ACR/EULAR score and some of the five criteria that comprise this score. In addition, some evidence for correlation between rheumatoid factor and Anti-Ro/SSA + and Anti-Ro/SSB+, and the proportion of B and NK cells were both correlated with focus score (Supplementary Fig. 3).

**Table 1:** Participant characteristics by case status, cluster membership, and case subgroup 2–4 compared to case subgroup 1–3

	Case	Non-case	*p*-value	Cluster 1	Cluster 2	Cluster 3	Cluster 4	*p*-value	Case subgroup 1–3	Case subgroup 2–4	*p*-value
***N***	592	467	-	343	342	326	48	-	234	358	-
Socio-demographic Characteristics											
Age (SD)	51.52 (13.57)	54.86 (13.09)	< 0.001	59.72 (10.41)	53.04 (13.84)	45.35 (11.82)	56.46 (13.60)	< 0.001	49.94 (13.68)	52.55 (13.41)	0.022
Race (%)											
Asian	320 (54.1)	140 (30.0)	< 0.001	99 (28.9)	189 (55.3)	136 (41.7)	36 (75.0)	< 0.001	113 (48.3)	207 (57.8)	0.028
White	272 (45.9)	327 (70.0)		244 (71.1)	153 (44.7)	190 (58.3)	12 (25.0)		121 (51.7)	151 (42.2)	
Smoking history (%)											
Current	21 (3.5)	68 (14.6)	< 0.001	47 (13.7)	9 (2.6)	33 (10.1)	0 (0.0)	< 0.001	14 (6.0)	7 (2.0)	0.005
Former	160 (27.0)	130 (27.8)		113 (32.9)	85 (24.9)	84 (25.8)	8 (16.7)		72 (30.8)	88 (24.6)	
Never	411 (69.4)	269 (57.6)		183 (53.4)	248 (72.5)	209 (64.1)	40 (83.3)		148 (63.2)	263 (73.5)	
2016 ACR/EULAR Criteria Items											
LSG with focal lymphocytic sialadentitis and focus score ≥ 1 (%)	478 (82.4)	19 (4.1)	< 0.001	84 (24.5)	297 (89.2)	94 (29.0)	22 (46.8)	< 0.001	161 (69.4)	317 (91.1)	< 0.001
Anti-Ro/SSA + (%)	479 (81.0)	23 (4.9)	< 0.001	74 (21.6)	284 (83.3)	116 (35.6)	28 (58.3)	< 0.001	170 (72.6)	309 (86.6)	< 0.001
Ocular staining score ≥ 5 on at least one eye (%)	471 (79.8)	145 (31.2)	< 0.001	129 (37.9)	309 (90.6)	144 (44.3)	34 (70.8)	< 0.001	139 (59.7)	332 (93.0)	< 0.001
Schirmer ≤ 5 mm/5min on at least one eye (%)	357 (60.6)	165 (35.4)	< 0.001	143 (41.9)	233 (68.5)	113 (34.7)	33 (68.8)	< 0.001	106 (45.5)	251 (70.5)	< 0.001
Unstimulated whole saliva flow rate ≤ 0.1 ml/min (%)	408 (68.9)	213 (45.6)	< 0.001	190 (55.4)	273 (79.8)	134 (41.1)	24 (50.0)	< 0.001	127 (54.3)	281 (78.5)	< 0.001
SjD (%)	-	-	-	107 (31.2)	328 (95.9)	127 (39.0)	30 (62.5)	< 0.001	-	-	-
Other SjD-related phenotypic features											
ACR/EULAR score; Mean (SD)	6.94 (1.84)	1.39 (0.99)	< 0.001	2.73 (2.37)	7.48 (1.91)	3.13 (2.61)	5.02 (2.86)	< 0.001	5.83 (1.72)	7.66 (1.54)	< 0.001
Focus score; Mean (SD)	2.87 (2.32)	0.26 (0.86)	< 0.001	0.65 (1.11)	3.71 (2.51)	0.82 (1.40)	1.36 (1.59)	< 0.001	1.63 (1.43)	3.70 (2.44)	< 0.001
ANA at 1:320 (%)	347 (58.6)	57 (12.2)	< 0.001	71 (20.7)	237 (69.3)	76 (23.3)	20 (41.7)	< 0.001	99 (42.3)	248 (69.3)	< 0.001
IGG, mg / dL; Mean (SD)	1779 (778)	1049 (352)	< 0.001	1118 (454)	1976 (813)	1240 (493)	1641 (807)	< 0.001	1435 (572)	2002 (813)	< 0.001
Complement Component 3, mg / dL; Mean (SD)	115.32 (26.69)	119.90 (31.73)	0.011	121.70 (29.21)	112.29 (23.58)	118.16 (33.63)	116.54 (26.12)	< 0.001	119.31 (30.28)	112.72 (23.76)	0.003
Complement Component 4, mg / dL: Mean (SD)	23.65 (9.41)	27.52 (9.09)	< 0.001	27.19 (8.64)	22.17 (8.24)	26.64 (10.67)	26.17 (9.13)	< 0.001	25.83 (10.52)	22.23 (8.32)	< 0.001
Anti-La/SSB+ (%)	309 (52.3)	10 (2.1)	< 0.001	33 (9.6)	222 (65.1)	49 (15.0)	15 (31.2)	< 0.001	72 (30.8)	237 (66.4)	< 0.001
Rheumatoid factor (%)	356 (60.1)	53 (11.3)	< 0.001	59 (17.2)	257 (75.1)	72 (22.1)	21 (43.8)	< 0.001	85 (36.3)	271 (75.7)	< 0.001
Max ocular SICCA score; Mean (SD)	7.56 (3.37)	3.57 (2.78)	< 0.001	4.09 (3.22)	8.76 (2.77)	4.37 (3.12)	6.56 (3.17)	< 0.001	5.50 (3.48)	8.89 (2.53)	< 0.001
Presence of germinal center (%)	112 (19.0)	5 (1.1)	< 0.001	12 (3.5)	90 (26.5)	13 (4.0)	2 (4.2)	< 0.001	20 (8.6)	92 (25.9)	< 0.001
Right parotid gland enlargement (%)	111 (18.8)	69 (14.8)	0.104	50 (14.6)	74 (21.6)	45 (13.8)	11 (22.9)	0.017	31 (13.2)	80 (22.3)	0.008
Left parotid gland enlargement (%)	113 (19.1)	68 (14.6)	0.063	49 (14.3)	73 (21.3)	44 (13.5)	15 (31.2)	0.001	31 (13.2)	82 (22.9)	0.005
Dry mouth symptoms (%)	537 (90.7)	413 (88.4)	0.268	319 (93.0)	317 (92.7)	272 (83.4)	42 (87.5)	< 0.001	206 (88.0)	331 (92.5)	0.095
Dry eye symptoms (%)	480 (81.1)	389 (83.3)	0.394	290 (84.5)	284 (83.0)	259 (79.4)	36 (75.0)	0.187	183 (78.2)	297 (83.0)	0.181

### Cell-type estimation in LSGs

**Fig. 2:**
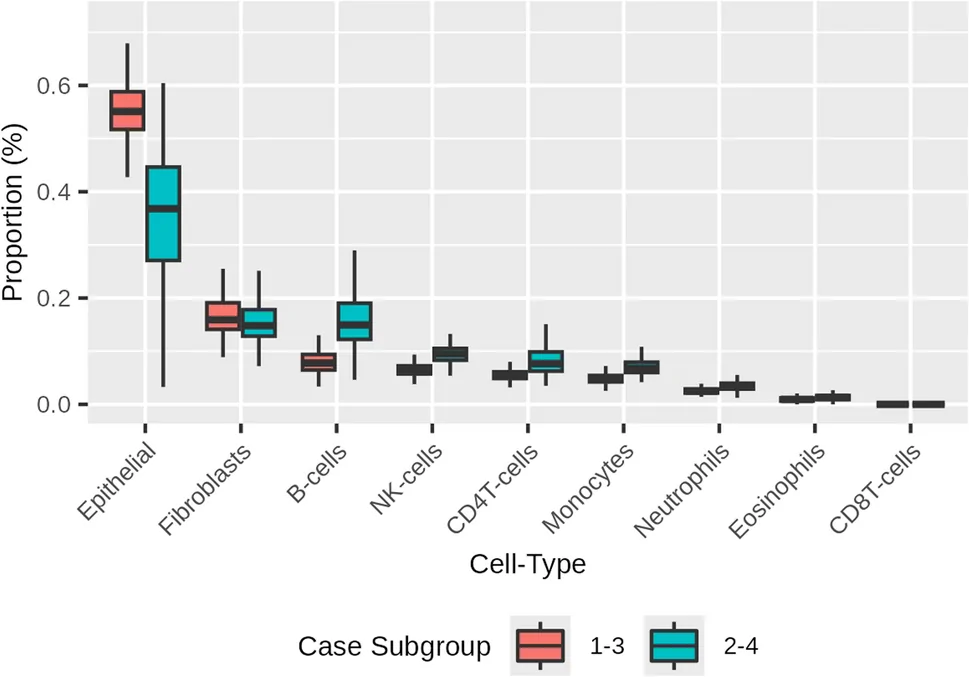
Estimated cell-type proportions in SjD case subgroup 1–3 compared to case subgroup 2–4. Cell-types are ordered from left to right from most abundant to least abundant. *P*-values are generated from t-tests

Cell-type estimation using HEpiDISH uses a reference of epithelial, fibroblast, and immune cells designed to estimate cell-types in solid tissues [28]. The reference does not consist of salivary gland cells and is rather, made to be used in generic solid tissues. Although results have been validated in multiple tissue types, it is not known how well estimation will perform in LSGs. Results of cell-type estimation showed epithelial cells to be the most prevalent cell-type overall, followed by fibroblasts, B-cells, NK cells, CDT + T cells, monocytes, neutrophils, eosinophils, and CD8 + T cells. Notably, cell-type composition differed between subgroup 2–4 and subgroup 1–3 (Fig. 2). Overall, subgroup 1–3 had a higher proportion of epithelial cells compared to immune cells, while immune cell proportions were increased in subgroup 2–4. The largest differences were observed between epithelial cells and B-cells. Among subgroup 1–3 these cell-types made up 55% and 8% of cells, respectively, while among subgroup 2–4, they made up 35% and 16%, respectively.

### Differential methylation distinguishes SjD case clusters

**Fig. 3:**
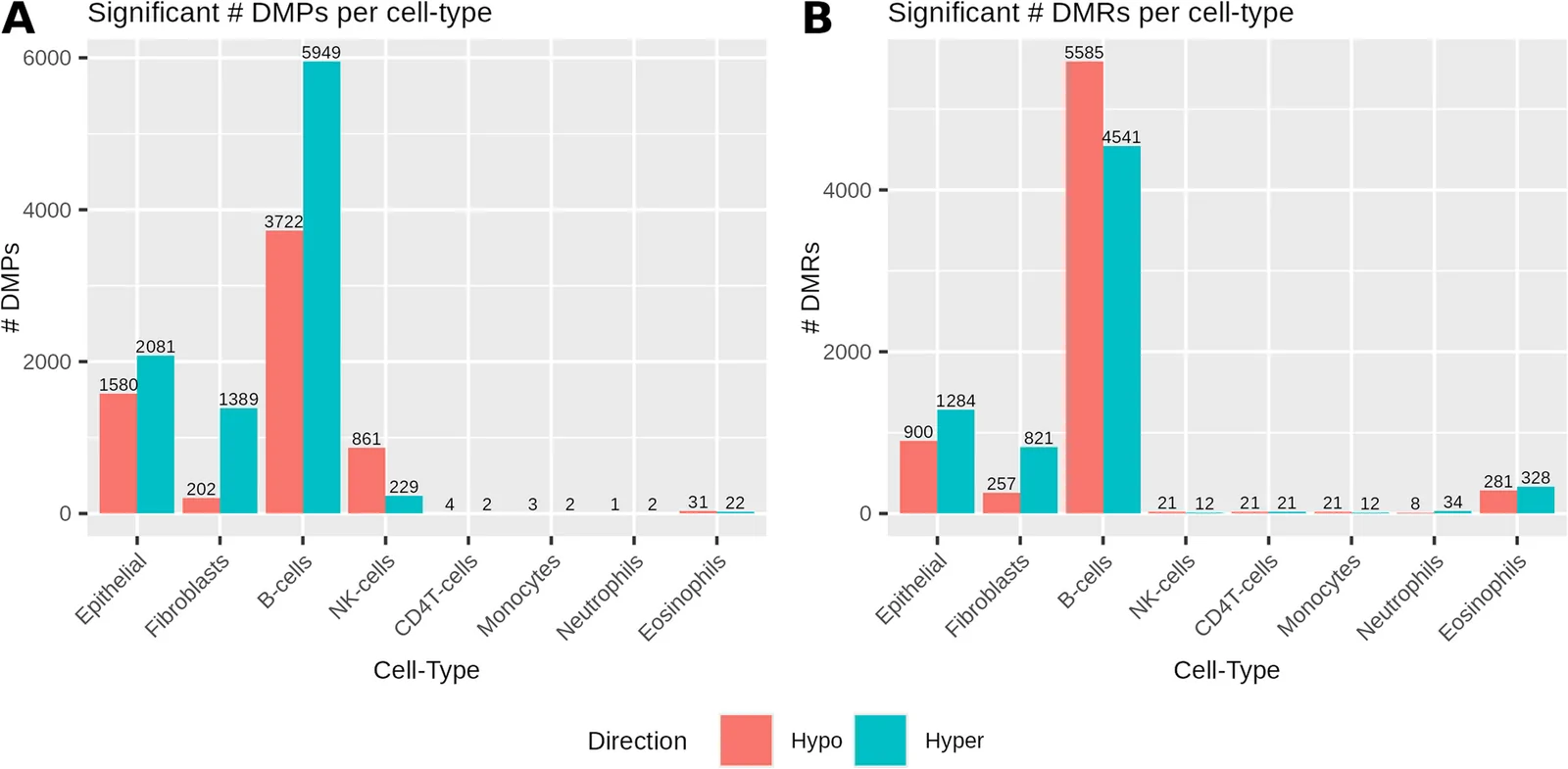
**A** Significant DMPs per cell-type between case subgroup 2–4 compared to case subgroup 1–3 and (**B**) Significant DMRs per cell-type between cases in subgroup 2–4 and subgroup 1–3. Cell-types are ordered from least abundant to most abundant. Case subgroup 1–3 is the reference group

To further understand the underlying DNA methylation differences between subgroup 2–4 and subgroup 1–3, we conducted differentially methylated position and regional analyses. In analyses of LSGs, we identified 815 significant DMRs before adjusting for cell-type proportions; however, after adjustment none remained significant. Since cell-types play specific roles in SjD pathology and had significantly different proportions between subgroup 2–4 compared to subgroup 1–3, these analyses were conducted using *CellDMC* to estimate the methylation differences at positions within each individual cell-type, and comb-p to estimate regional differences. Figure 3 summarizes significant DMP (Bonferroni *p* < 0.05) and DMR (Sidak *p* < 0.05) results. We identified a total of 16,996 DMRs across all cell-types distinguishing the two subgroups. The highest number of significant DMPs and DMRs were observed in B-cells, followed by epithelial cells. All significant DMP and DMR results for B-cells and epithelial cells are summarized in Supplementary Tables 1–4.

For the comparison of subgroups 2–4 to 1–3, some of the top DMRs in B-cells were located in genes including *NR2F2* (56% decrease in methylation), *PPP1R18* (93% decrease in methylation), *NDRG2* (95% increase in methylation), and *LAT* (75% decrease in methylation), all of which were located within the gene body. *NR2F2* encodes a member of the steroid thyroid hormone superfamily of nuclear receptors and encodes a transcription factor which regulates multiple genes [41]. *PPP1R18* is involved in actin and phosphatase binding [42]. *NDRG2* plays a role in neurite outgrowth and is broadly expressed in salivary glands [43]. *LAT* plays a role in immune responses as part of the T-cell antigen receptor signaling pathway [44]. Top DMRs in epithelial cells were located in genes including *IRF5* (8% decrease in methylation), *ARID3A* (12% decrease in methylation), and *ZMYND8* (2% increase in methylation). *IRF5* encodes a protein in the interferon regulatory pathway and is implicated in many autoimmune diseases [45]. *ARID3A* encodes a DNA binding protein involved in cell-lineage and cell-cycle control [46]. *ZMYND8* encodes a protein involved in the development of lymphoma [47]. 

### Gene set enrichment analysis

**Fig. 4:**
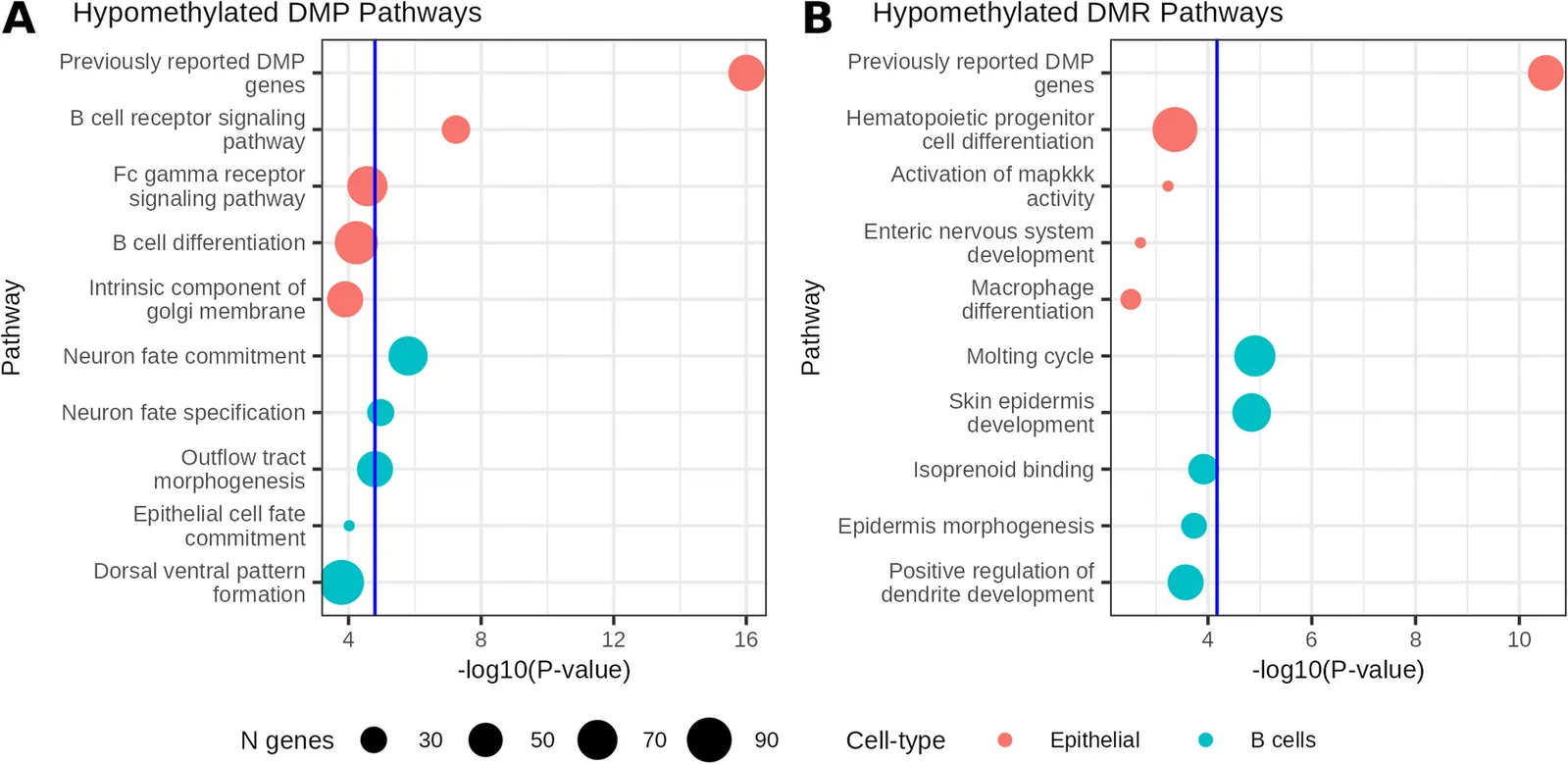
**A** Hypomethylated DMP pathways in Epithelial and B-cells, (**B**) Hypomethylated DMR pathways in Epithelial and B-cells. Circle size denotes the number of genes in the pathway, color indicates cell-type, and the blue line denotes FDR significance. Case subgroup 1–3 is the reference group

GSEA of DMRs located inside genes and in gene promoters showed evidence of enrichment of previously identified genes in human pathways which are *hypomethylated* in SjD cases vs. non-cases in epithelial cells (Fig. 4a). Since epithelial cells were the most prevalent cell-type in these samples, epithelial cell methylation profiles contribute the most to the LSG methylation profiles that have been previously studied. In B-cells, pathways related to the molting cycle (referring to the periodic casting off and regeneration of an outer covering of cuticle, hair, skin), as well as skin epidermis development, contained *hypomethylated* DMRs. In epithelial cells, pathways including lens morphogenesis and vocalization behavior were enriched for *hypermethylated* DMRs (Supplementary Fig. 2). Similarly, epithelial cell DMPs were enriched in previously identified genes *hypomethylated* in SjD cases compared to controls (Fig. 4b). DMPs were also enriched in pathways for B-cell receptor signaling in epithelial cells. In B-cells, pathways were also enriched for neuron commitment and fate. No pathways were enriched with *hypermethylated* DMPs in any cell-type (Supplementary Fig. 2). All top results for GSEA analysis are summarized in Supplementary Tables 5–12.

## Discussion

We used DNA methylation data from LSGs to identify clinically relevant clusters of participants with distinct methylation profiles. Subgroup 2–4 demonstrated significantly more phenotypic features that reflected higher levels of autoimmunity, and a higher frequency of SjD-related clinical manifestations compared to subgroup 1–3. These subgroups were also characterized by different proportions of estimated cell-types with subgroup 2–4 exhibiting a greater proportion of immune cells (and lower proportions of epithelial cells) to subgroup 1–3. These findings provide greater insight into SjD pathogenesis and identify biological differences which may contribute to clinical heterogeneity.

Cell-types play different roles in SjD pathogenesis and are increasingly well understood [10]. Previous studies of DNA methylation in SjD have shown that cases and controls have different methylation profiles in specific cell-types with little overlap of differential methylation and have suggested that cell-specific analyses are necessary to understand epigenetic contributions to SjD [14]. Our results highlight large differences in cell-type proportions between our two SjD subgroups, indicating immune cells are increased in subgroups with phenotypic features reflecting higher levels of autoimmunity, and more SjD-related clinical manifestations. This finding is also in agreement with recorded focus scores for these cases. This score is a measure of immune cell infiltration in LSGs. The focus score was significantly higher (3.7 compared to 1.6) in subgroup 2–4 compared to subgroup 1–3. The majority of our findings were in epithelial cells and B-cells, both of which play a major role in SjD pathogenesis [10]. In SjD, salivary gland epithelial cells are a source of inflammatory cytokines and are sensitive to toll-like receptor apoptosis. The expression of cytokines by epithelial cells contributes to the formation of germinal centers. Epithelial cells can additionally lead to increased B-cell activating factor (BAFF), which can stimulate B-cells [10]. *Hypomethylated* DMPs in epithelial cells were enriched in B-cell receptor signaling pathways and could indicate that epithelial cells in subgroup 2–4 are contributing to the observed increased B-cell proportions. B-cells drive reduced salivary flow rate, increased lymphocytic infiltration of salivary glands, formation of autoantibodies, and the formation of germinal centers [10]. B-cell DMPs were enriched in pathways related to neuron fate commitment. Neurons in salivary glands contribute towards signaling saliva production and there is debate whether SjD may involve neurologic damage to the salivary gland which in turn triggers inflammatory responses [48]. Previous studies have also shown enrichment of methylation differences in neuronal pathways [21]. B-cell DMRs were enriched in pathways for molting cycles and skin epidermis development. Interestingly, chitinases, which are gaining attention as key players in the innate immune response, have been shown to be increased in more inflamed tissue of subjects with more clinically severe SjD [49]. 

The most significant DMR in B-cells with a 56% decrease in methylation in subgroup 2–4, was located in *NR2F2*, a gene previously reported to show differential expression in lacrimal glands of mouse models with SjD compared to controls [50]. A DMR in *PPP1R18* has previously had individual CpG sites identified as differentially methylated in SLE cases compared to controls [51]. SjD and SLE share many clinical features–while our study was restricted to subjects with primary SjD, this disorder can often be diagnosed among individuals with other autoimmune diseases, such as SLE and RA [52]. Estrogen deficiency is known to lead to oral dryness, and previous studies have shown introduction of the *NDRG2* gene, which is involved in estrogen-mediated ion and fluid transport, greatly increases saliva production in rat models [53]. Since SjD is more common in women than men, it is thought that estrogen levels may play a role in disease pathogenesis. Estrogen can influence the immune system through both immunosuppressive and immunoenhancing effects, depending on the context and levels. Changes in estrogen levels, such as those occurring during menopause, might alter immune system regulation, potentially contributing to the onset or worsening of SjD [54]. 

Interestingly, increased estrogen shows a protective effect in mouse models of SjD, though the side effects of estrogen supplementation limit therapeutic potential [53, 55]. We observed a 95% increase in methylation in a DMR located in *NDRG2*, which could indicate decreased expression in subgroup 2–4 compared to those in subgroup 1–3. *LAT* is a gene involved in activation of the T-cell antigen receptor (TCR) signal transduction pathway [44]. A study examining germline genetics in familial RA, SLE, and SjD reported evidence for variation within *LAT* and other TCR signaling pathway genes associated with these autoimmune diseases in families [56]. We observed a 75% decrease in methylation in a DMR located in *LAT* when subgroup 2–4 were compared with those in subgroup 1–3.

In epithelial cells, *IRF5* contained a DMR with an 8% decrease in methylation in subgroup 2–4 compared to those in subgroup 1–3. Associations with polymorphisms in *IRF5* have been reported in both SjD and SLE [57]. Dysregulation of interferon, commonly referred to as an “interferon signature,” is a hallmark feature of SjD and has been observed in both salivary glands and blood [58]. *ARID3A* contained a DMR with a 12% decrease in methylation in subgroup 2–4 compared to those in subgroup 1–3. Prior studies in PBMC B-cells of patients with SLE have shown that expression of *ARID3A* is greatly increased in SLE cases compared to controls and is associated with more severe SLE within cases [59]. *ZMYND8*, which contained a DMR with 2% increased methylation in subgroup 2–4, is a tumor suppressor gene involved in the development of lymphoma. Participants with SjD are at an overall increased risk of non-Hodgkin’s lymphoma [60]. *Hypermethylation* could indicate decreased expression of the tumor suppressor gene, and potentially increased risk for development of lymphoma.

Our study has several important strengths. While many methylation analyses are performed using whole blood because of the ease of sample collection and processing, we were able to use LSGs, which facilitated methylation analysis of the primary target tissue for SjD. Previous analyses of LSGs have not adjusted for cell-type differences. Our study demonstrates this is an important adjustment in the assessment of LSGs in SjD. This is the largest sample size for a methylation study of LSGs in SjD to-date and we observed statistically significant results. Our unique study design included symptomatic, and therefore informative, non-cases from the SICCA registry to increase our sample size for assigning clusters.

One limitation of this study is the lack of *specific* methylation reference panels for LSGs. Existing resources, while informative, do not yet provide comprehensive coverage or validation for all relevant cell-types. Nonetheless, cell-type proportions were estimated using a state-of-the-art reference composed of epithelial, fibroblasts, and immune cells, together with the methodology applied in HEpiDISH [28]. It is possible that some of the estimated proportions and cell-specific methylation profiles generated in this analysis may be spurious; therefore, future work should aim to replicate these findings using cell-sorted methylation data. For the future use of bulk LSG data, these estimates can also be improved when reference panels are developed for LSGs and should be used to replicate the reported results. Additionally, these panels should include more granular cell-type estimation. Given the heterogeneity of salivary gland tissue, future studies should also consider the spatial context of the cellular heterogeneity, which was not possible with our data. Another limitation of the study was that CD8 + T cells were not identified in higher proportions in the LSGs. This was not as expected, given what is known about SjD disease pathogenesis [61, 62]. There appeared to be high correlation between reference DNA methylation values distinguishing NK, CD8 + and CD4 + T cells so the algorithm may not reliably distinguish these cell-types. Our results suggest that these cells were indeed present but not reliably categorized. We focused our study findings on both B cells and epithelial cells for which confidence for correct cell proportion assignment was high. Future studies should incorporate DNA methylation profiles derived from sorted cells or single cell analysis. Despite this limitation, the CellDMC method developed by Zheng et al. and applied in this study has been shown to outperform reference-based and reference-free methodologies for differentially methylated cell-type analysis in bulk tissues [35]. Additionally, effect sizes for DMRs were determined using linear regression with beta-values. This can result in beta coefficients corresponding to a greater than 100% difference in methylation due to adjustment of covariates, which is not biologically plausible [63]. The method implemented in this analysis truncated regression coefficients ≥ 1 and ≤ -1 to 1 and − 1 respectively. Methods which allow for retaining the intercept, which would allow for M-value differences to be back-transformed into biologically plausible beta value differences should be implemented in the future [63]. While many of the top findings demonstrated evidence for large deltas in methylation differences, many CpGs had small differences. While there is no agreed upon value of methylation difference which is considered biologically (or clinically) significant, it is possible that lower delta changes may not significantly impact human health or disease. Only female participants were included in this study to reduce heterogeneity, and although SjD is much more prevalent in females than males, males exhibit different clinical patterns of disease, and these results may not be generalizable to all individuals with SjD [54]. Additionally, this study was limited to individuals of European and East Asian ancestry to reduce heterogeneity and may not be generalizable to other populations. Finally, the SICCA cohort was collected prior to development and validation of the EULAR Sjogren’s syndrome disease activity index (ESSDAI), which is now used to measure systemic disease activity, and would have allowed for further assessment of phenotypic heterogeneity within the clusters.

In summary, we used DNA methylation data from LSGs to cluster SjD cases and non-cases from the SICCA registry, resulting in distinct groups of SjD cases with different clinical characteristics. Two observed subgroups of SjD cases exhibited different cell-type proportions and differential methylation within predicted cell-types. These findings highlight the need to perform cell-specific analysis in SjD. We describe genes and pathways with significant differences in DNA methylation, which may inform our understanding of clinical heterogeneity in SjD, establish sites within the genome that can serve as biomarkers of prognosis and progression, and provide future targets for therapeutic intervention.

## Supplementary Information

Supplementary Material 1.

Supplementary Material 2: FigS1. VAE training loss. FigS2. (A) Hypermethylated DMP pathways in Epithelial and B-cells, (B) Hypermethylated DMR pathways in Epithelial and B-cells. Circle size denotes the number of genes in the pathway, color indicates cell-type, and the blue line denotes FDR significance. Case subgroup 1-3 is the reference group. FigS3. Correlations among clinical features and cell-type proportions.

## Data Availability

The dataset supporting the conclusions of this article is available through GEO, [Accession number TBD].
