# Correction: Sjögren’s patient subgroups identified through whole genome DNA methylation profiling

**DOI:** 10.1186/s13075-026-03883-x

**Published:** 2026-08-22

**Authors:** Olivia Solomon, Caroline Shiboski, Kimberly E. Taylor, Hong Quach, Diana Quach, Lisa F. Barcellos, Lindsey A. Criswell

**Affiliations:** 1 Genetic Epidemiology and Genomics Laboratory, School of Public Health, University of California, 324 Stanley Hall, MC#3220, Berkeley, CA 94720 USA; 2 Center for Computational Biology, Division of Computing, Data Science and Society, University of California, Berkeley, CA USA; 3 Department of Orofacial Sciences, School of Dentistry, University of California, San Francisco, CA USA; 4 Department of Medicine, Russell/Engleman Rheumatology Research Center, University of California, San Francisco, CA USA; 5 Genomics of Autoimmune Rheumatic Disease Section, National Human Genome Research Institute, National Institute of Health, Bethesda, MD USA

**Correction: Arthritis Res Ther 28**,** 41 (2026)**

**https://doi.org/10.1186/s13075-026-03744-7**

Following publication of the original article [1], the authors reported a missing word in Abstract: Methods section.

The incorrect statement in Methods section is:

**LSGs from 1**,**059** SjD cases (*n* = 592) and symptomatic non-cases (*n* = 467) were profiled using the Illumina HumanMethylationEPIC BeadChip, and cell-type proportions were estimated using a solid-tissue reference.

The correct statement should be:

**LSGs from 1**,**059 individuals**, SjD cases (*n* = 592) and symptomatic non-cases (*n* = 467), were profiled using the Illumina HumanMethylationEPIC BeadChip, and cell-type proportions were estimated using a solid-tissue reference.

The original article [1] has been updated.
