# Supplementary figures and images for: Sjögren’s patient subgroups identified through whole genome DNA methylation profiling
Source: Arthritis Res Ther. 2026 Feb 4;28:41. doi: 10.1186/s13075-026-03744-7 (PMC12896019; doi:10.1186/s13075-026-03744-7)

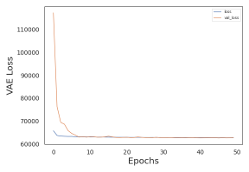

Supplement: Supplementary file 2 — Supplementary Material 2: FigS1. VAE training loss. FigS2. (A) Hypermethylated DMP pathways in Epithelial and B-cells, (B) Hypermethylated DMR pathways in Epithelial and B-cells. Circle size denotes the number of genes in the pathway, color indicates cell-type, and the blue line denotes FDR significance. Case subgroup 1-3 is the reference group. FigS3. Correlations among clinical features and cell-type proportions. [file 13075_2026_3744_MOESM2_ESM.zip › sjd_sup_figure_1.png]

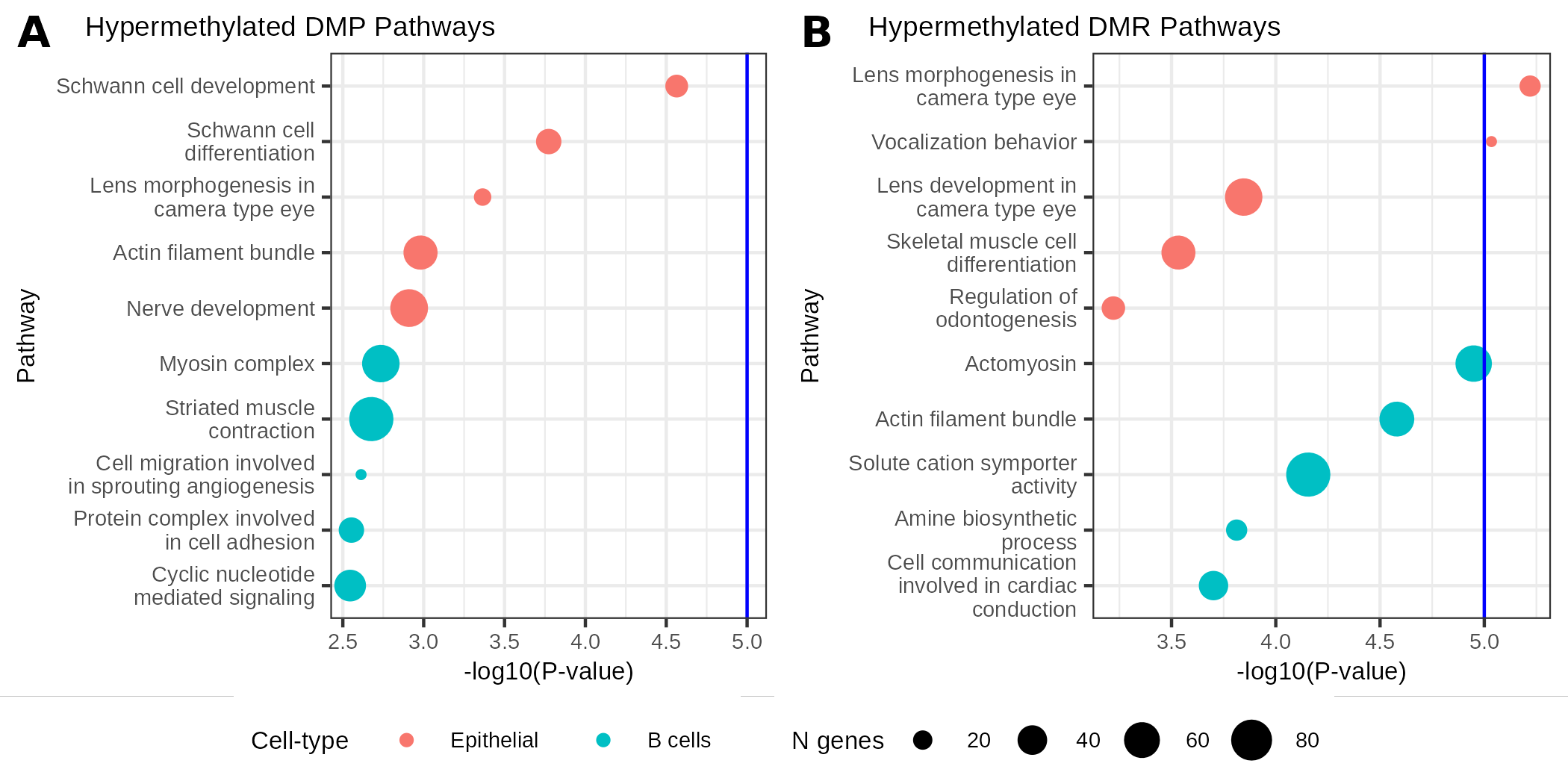

Supplement: Supplementary file 2 — Supplementary Material 2: FigS1. VAE training loss. FigS2. (A) Hypermethylated DMP pathways in Epithelial and B-cells, (B) Hypermethylated DMR pathways in Epithelial and B-cells. Circle size denotes the number of genes in the pathway, color indicates cell-type, and the blue line denotes FDR significance. Case subgroup 1-3 is the reference group. FigS3. Correlations among clinical features and cell-type proportions. [file 13075_2026_3744_MOESM2_ESM.zip › sjd_sup_figure_2.tiff]

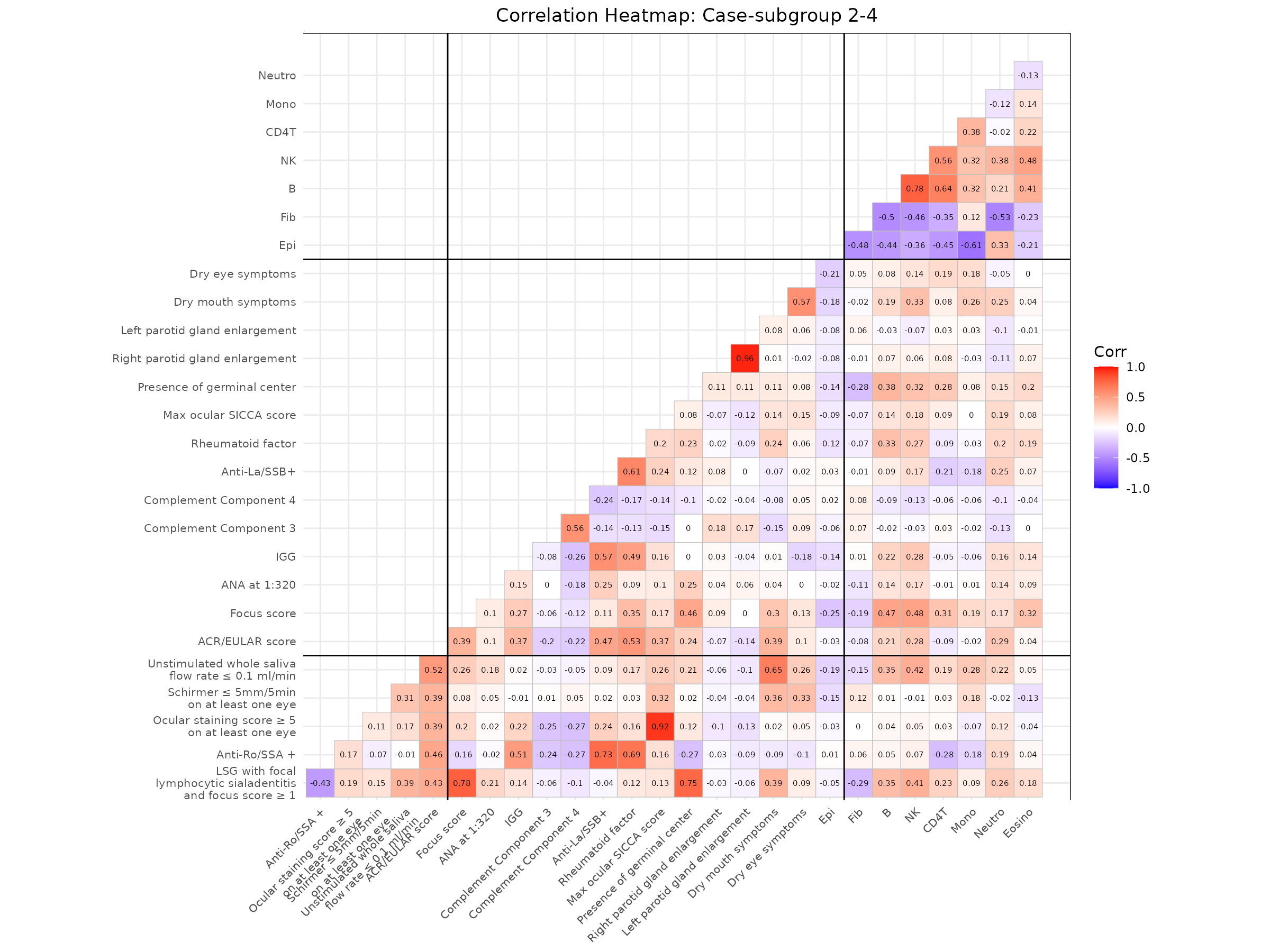

Supplement: Supplementary file 2 — Supplementary Material 2: FigS1. VAE training loss. FigS2. (A) Hypermethylated DMP pathways in Epithelial and B-cells, (B) Hypermethylated DMR pathways in Epithelial and B-cells. Circle size denotes the number of genes in the pathway, color indicates cell-type, and the blue line denotes FDR significance. Case subgroup 1-3 is the reference group. FigS3. Correlations among clinical features and cell-type proportions. [file 13075_2026_3744_MOESM2_ESM.zip › sjd_sup_figure_3.png]
